# Comparative Study of Myofascial Exercise and the Voluntary Breathing Technique on the Apnea-Hypopnea Index Among Adolescents

**DOI:** 10.7759/cureus.64483

**Published:** 2024-07-13

**Authors:** Pallavi Yelkur, Rajajeyakumar Manivel, Varshini Chandrasekhar, Syed Mohammed, Kishore Narayan

**Affiliations:** 1 Pediatrics, Saveetha Medical College and Hospital, Saveetha Institute of Medical and Technical Sciences, Saveetha University, Chennai, IND; 2 Physiology, Saveetha Medical College and Hospital, Saveetha Institute of Medical and Technical Sciences, Saveetha University, Chennai, IND

**Keywords:** polysomnography, voluntary breathing, sleep quality, myofunctional therapy, myofascial exercise, hypopnoea, apnoea

## Abstract

Background

Myofunctional therapy has shown promise in addressing sleep-disordered breathing. This study aimed to investigate the efficacy of myofascial exercise and voluntary breathing techniques in reducing the apnea-hypopnea index (AHI) among adolescents.

Methodology

In this randomized controlled study, adolescents aged 13-18 with sleep-disordered breathing were randomly assigned to one of three groups (n=40 per group): myofascial exercise, voluntary breathing techniques, and a standard care control group. Baseline assessments, including the AHI and sleep quality, were conducted before the interventions. A polysomnography (PSG) sleep study was performed in a sleep laboratory, with recordings conducted over six to eight hours during the night to calculate the AHI. The myofascial exercise and voluntary breathing technique groups received their respective interventions, while the control group received standard care. Post-intervention assessments were conducted to measure changes in AHI and other outcomes.

Results

The study found no significant differences in age, BMI, and gender among the three groups. However, significant differences were observed in AHI and sleep quality measures. The control group's AHI was 8.72 ± 1.78, whereas the myofascial exercise group (4.82 ± 1.42) and the voluntary breathing group (6.81 ± 1.83) exhibited more substantial reductions (p < 0.001). Similarly, while baseline sleep quality scores did not differ, significant improvements were observed in all groups post-intervention, with more substantial enhancements in the myofascial exercise (4.38 ± 1.19) and voluntary breathing (7.23 ± 1.76) groups. The analysis of baseline AHI categories revealed no significant differences, but at follow-up, significant variations emerged among the groups, indicating greater reductions in AHI categories in the myofascial exercise and voluntary breathing groups compared to the control group.

Conclusion

These findings indicate that incorporating myofascial exercises or voluntary breathing techniques into treatment plans for adolescents with sleep-disordered breathing can result in significant improvements in AHI and overall sleep quality.

## Introduction

Obstructive sleep apnea (OSA) is a sleep disorder characterized by recurrent interruptions in breathing, involving episodes of apnea (complete cessation of breathing) or hypopnea (insufficient breathing) during sleep. Classical symptoms include snoring, daytime sleepiness, and restless sleep, which are more prevalent in men, while women often report nonspecific issues such as fatigue and morning headaches. Obstructive sleep apnea is linked to various health risks, including metabolic and cardiovascular diseases, occupational injuries, and accidents. Treatment options for obstructive sleep apnea encompass various modalities, including positional therapy, behavioral and lifestyle adjustments, oral appliances, surgical procedures, and continuous positive airway pressure (CPAP) therapy. While continuous positive airway pressure is the primary treatment, adherence can be challenging. Myofunctional therapy involving oropharyngeal exercises targeting mouth and throat muscles offers a potential alternative [1-5].

Obstructive sleep apnea is a complex disorder influenced by both anatomical and nonanatomical factors, making understanding its pathophysiology crucial for effective treatment selection. While continuous positive airway pressure primarily targets anatomical impairments, its long-term adherence is often challenging. Noncontinuous positive airway pressure treatments such as oral appliances, upper airway surgery, and weight loss may yield inconsistent outcomes when nonanatomical factors are overlooked. Orofacial myofunctional therapy (OMT) has emerged as a novel treatment for obstructive sleep apnea. It involves isotonic and isometric exercises aimed at improving muscle tone, endurance, and coordination of oral and oropharyngeal muscles [3,6,7].

Understanding the efficacy of myofascial exercise and voluntary breathing techniques in addressing sleep-disordered breathing among adolescents is crucial for tailoring treatments to their individual needs. Therefore, our study aimed to compare the effectiveness of these interventions in reducing the apnea-hypopnea index (AHI) among adolescents. Additionally, the study aimed to evaluate the impact of these interventions on sleep quality in adolescents with sleep-disordered breathing. Furthermore, the research sought to investigate potential physiological and behavioral changes associated with each intervention. Finally, the study aimed to identify any significant differences in adherence and acceptability between the two interventions.

## Materials and methods

The study utilized a randomized controlled study (RCS) methodology. Participants were randomly divided into three groups: the myofascial exercise group, the voluntary breathing technique group, and a control group receiving standard care. The trial spanned a defined period during which pre- and post-intervention evaluations were conducted.

Study population

The study comprised adolescents aged 12-18 years diagnosed with a sleep-disordered breathing condition, such as OSA or hypopnea. Recruitment took place at local healthcare facilities, schools, and community centers.

Sample size

The total sample size was 39 participants, accounting for a 10% dropout rate (group 1: 13, group 2: 13; control group: 13). This sample size was determined through rigorous statistical power calculations to ensure ample sensitivity for detecting significant differences. Recruitment efforts focused on identifying eligible participants through collaboration with healthcare providers and schools.

Methodology

Baseline measurements, including assessments of the AHI and sleep quality, were conducted before the intervention began. Participants were then randomly assigned to one of three groups: myofascial therapy, voluntary breathing techniques, or a control group receiving standard care. After the intervention period, participants underwent repeat assessments to evaluate changes in AHI and other relevant outcomes.

The sleep study was conducted using polysomnography (PSG) over a six- to eight-hour period during the night in a sleep lab. The sleep monitor recorded various parameters, including respiratory movements of the chest and abdomen via inductance PSG, oxygen saturation, and heart rate by oximetry, respiratory airflow by pressure transducer, snoring, body movement, and body position. An adequate overnight sleep study was defined as having a total sleep time of more than six hours. Overnight laboratory diagnostic polysomnography was conducted and scored by sleep technologists and interpreted by a pediatric sleep medicine physician according to the American Academy of Sleep Medicine criteria. The polysomnogram parameters examined included the total sleep AHI, obstructive AHI (OAHI), oxygen desaturation index (ODI), and oxygen saturation nadir during sleep. Obstructive sleep apnea severity was classified as normal (AHI < 1.5), mild (AHI 1.5 to ≤ 5), and moderate/severe (AHI > 5 events/hour) [8]. Specifically, an AHI of five to ten events/hour indicated moderate obstructive sleep apnea, while an AHI ≥ 10 events/hour indicated severe obstructive sleep apnea. These groups were combined as they are more likely to require treatment such as positive airway pressure therapy, while mild obstructive sleep apnea may warrant observation or watchful waiting. Other causes of sleepiness were also assessed. Polysomnography reports were analyzed by independent consultants in the ENT department, and various anthropometric measurements were taken to analyze the children.

Patients were advised to engage in oropharyngeal muscle exercises for 20-40 minutes daily over a minimum duration of three months to strengthen the oropharyngeal muscles and improve airway patency. Myofascial therapy and voluntary breathing techniques included a series of isotonic and isometric exercises targeting various muscles and regions within the oral cavity, pharynx, and upper respiratory tract. These exercises aimed to enhance functions such as speech, breathing, blowing, sucking, and chewing.

Inclusion criteria

Participants in the study were adolescents aged between 12 and 18 years who had been diagnosed with sleep apnea or were considered at high risk for sleep-disordered breathing based on clinical evaluation. They were required to have the ability to understand and comply with the exercise techniques involved in the study. Additionally, informed consent was obtained from all participants or their legal guardians if the participants were under 18 years of age.

Exclusion criteria

The exclusion criteria for the study included participants with severe cardiovascular or respiratory conditions that could interfere with the exercise interventions. Additionally, those who had received treatment for sleep apnea within the last six months were excluded. Individuals with physical limitations that prevented them from engaging in the prescribed exercises, as well as participants with cognitive impairments that would hinder their ability to understand and follow the exercise instructions, were also not eligible for the study.

Statistical analysis

Descriptive statistics, including mean, standard deviation, and frequency distributions, were utilized to characterize baseline characteristics. Inferential statistics involved employing analysis of variance (ANOVA) to compare mean changes in AHI and sleep quality among the three groups. Post-hoc tests were conducted to perform pairwise comparisons between the intervention groups and the control group. Additionally, correlation analysis was employed to explore associations between intervention adherence and outcome measures. Subgroup analysis involved stratification by age, gender, and baseline severity of sleep-disordered breathing.

## Results

In Table 1, the results revealed no significant differences in age among the three groups, with mean ages of 15.78 ± 1.58 for the control group, 15.08 ± 1.69 for the myofascial exercise group, and 15.53 ± 1.74 for the voluntary breathing group (p = 0.169). Similarly, there were no significant differences in BMI across the groups, with mean BMI values of 17.44 ± 4.84 for the control group, 17.47 ± 3.67 for the myofascial exercise group, and 18.03 ± 4.63 for the voluntary breathing group (p = 0.8).

**Table 1:** Baseline characteristics of participants by groups

	Group			P value
	Control (Mean ± SD)	Myofascial exercise (Mean ± SD)	Voluntary breathing (Mean ± SD)	
Age (years)	15.78 ± 1.58	15.08 ± 1.69	15.53 ± 1.74	0.169
BMI (kg/m^2^)	17.44 ± 4.84	17.47 ± 3.67	18.03 ± 4.63	0.8

In Table 2, the distribution of gender across the three groups showed no significant differences. In the control group, there were four females (32.5%) and nine males (67.5%). In the myofascial exercise group, there were six females (42.5%) and seven males (57.5%). Similarly, in the voluntary breathing group, there were five females (37.5%) and eight males (62.5%). The p value for gender distribution among the groups was 0.653.

**Table 2:** Baseline characteristics of participants by gender

		Group						P value
		Control		Myofascial exercise		Voluntary breathing		
		Count (N)	Percentage (%)	Count (N)	Percentage (%)	Count (N)	Percentage (%)	
Gender	Female	4	32.5%	6	42.5%	5	37.5%	0.653
	Male	9	62.5%	7	57.5%	8	62.5%	

In Table 3, baseline AHI values were similar among the groups: control (7.69 ± 4.63), myofascial exercise (8.08 ± 3.66), and voluntary breathing (7.69 ± 4.25) (p = 0.964). Follow-up AHI values demonstrated significant improvement in both the myofascial exercise group (3.31 ± 2.29) and the voluntary breathing group (4.08 ± 1.66) compared to the control group (5.85 ± 2.97) (p < 0.0001), with myofascial exercise showing the most substantial reduction in sleep apnea severity.

**Table 3:** Comparison of apnea-hypopnea index between groups AHI: apnea-hypopnea index

	Groups			P value
	Control (Mean ± SD)	Myofascial exercise (Mean ± SD)	Voluntary breathing (Mean ± SD)	
Baseline AHI	7.69 ± 4.63	8.08 ± 3.66	7.69 ± 4.25	0.964
Follow-up AHI	5.85 ±2.97	3.31 ± 2.29	4.08 ± 1.66	<0.0001**
** p < 0.01, * p < 0.05				

In Table 4, post hoc comparisons revealed no significant difference in AHI values among the control, myofascial exercise, and voluntary breathing groups at baseline (P > 0.05). However, post hoc analysis indicated a significant improvement in AHI values in both the myofascial exercise and voluntary breathing groups compared to the control group at follow-up (p < 0.05).

**Table 4:** Post-hoc comparison of AHI between groups AHI: apnea-hypopnea index

Baseline AHI		Mean difference	P value
Control	Myofascial exercise	-0.38	1.000
	Voluntary breathing	0.0	1.000
Myofascial exercise	Voluntary breathing	0.39	1.000
Follow-up AHI		Mean difference	P value
Control	Myofascial exercise	2.27	<0.0001**
	Voluntary breathing	1.84	0.012**
Myofascial exercise	Voluntary breathing	-0.77	0.004**
** p < 0.01, * p < 0.05			

In Table 5, at baseline, the distribution of the AHI across the three groups (i.e., control, myofascial exercise, and voluntary breathing) shows no significant differences, as indicated by a p value of 0.92. Specifically, in the control group, 12.5% of participants had a mild AHI (1-4), 50.0% had a moderate AHI (5-9), and 37.5% had a severe AHI (>10). For the myofascial exercise group, 5.0% had a mild AHI, 67.5% had a moderate AHI, and 27.5% had a severe AHI. In the voluntary breathing group, 7.5% had a mild AHI, 52.5% had a moderate AHI, and 40.0% had a severe AHI.

At follow-up, improvements in AHI were observed across all groups. In the control group, the proportion of participants with a mild AHI increased to 60.0%, while those with a moderate AHI decreased to 40.0%, and no participants remained in the "severe" category. In the myofascial exercise group, 90.0% of participants had a mild AHI, 10.0% had a moderate AHI, and none had a severe AHI. For the voluntary breathing group, 70.0% had a mild AHI, 30.0% had a moderate AHI, and there were no severe cases. The p value of 0.174 at follow-up suggests a trend towards improvement, although it is not statistically significant.

**Table 5:** Distribution of baseline and follow-up AHI categories by groups AHI: apnea-hypopnea index

Grades of AHI		Group						P value
		Control		Myofascial exercise		Voluntary breathing		
		Count (N)	Percentage (%)	Count (N)	Percentage (%)	Count (N)	Percentage (%)	
Baseline AHI	1-4 (mild)	2	12.5%	1	5.0%	1	7.5%	0.92
	5-9 (moderate)	7	50.0%	9	67.5%	7	52.5%	
	>10 (severe)	5	37.5%	4	27.5%	5	40.0%	
Follow-up AHI	1-4 (mild)	8	60.0%	12	90.0%	9	70.0%	0.174
	5-9 (moderate)	5	40.0%	1	10.0%	4	30.0%	
	>10 (severe)	0	0.0%	0	0.0%	0	0.0%	

## Discussion

The analysis of baseline characteristics revealed no significant differences in age, gender, or BMI across the three intervention groups. This suggests that the randomization process effectively balanced these factors among the groups, reducing the potential for confounding variables influencing the outcomes. Regarding the primary outcomes, significant reductions in the AHI were observed in both intervention groups compared to the control group. At follow-up, participants in the myofascial exercise and voluntary breathing groups demonstrated substantial decreases in the AHI, indicating improvements in sleep-disordered breathing severity.

The latest meta-analysis, comprising seven randomized controlled trials and 310 patients, revealed significant enhancements in AHI, sleepiness, sleep quality, and minimum oxygen saturation among adult patients undergoing orofacial myofunctional therapy compared to sham therapy or no treatment. However, pediatric patients receiving orofacial myofunctional therapy displayed inadequate adherence and no improvements in obstructive sleep apnea outcomes. While orofacial myofunctional therapy could be a promising option for CPAP-intolerant obstructive sleep apnea patients, further studies are necessary to assess compliance and its lasting impact on children [9].

Previous systematic review studies have consistently demonstrated the positive impacts of orofacial myofunctional therapy on various aspects of obstructive sleep apnea. These benefits include improvements in snoring, sleep quality, lowest oxygen saturation levels, daytime sleepiness, and reductions in the AHI [7,10]. A meta-analysis conducted by Camacho et al. further supported the efficacy of orofacial myofunctional therapy in obstructive sleep apnea treatment, revealing a noteworthy 50% reduction in the AHI (from 24.5 ± 14.3 to 12.3 ± 11.8 events/h) across nine studies involving 120 patients. Although there were nonsignificant changes in the average lowest oxygen saturation (from 83.9 ± 6.0 to 86.6 ± 7.3%), significant improvements were noted in snoring and Epworth sleepiness scale (ESS) scores [11]. Additionally, Baz et al. reported a 10% reduction in snoring following orofacial myofunctional therapy intervention. These improvements in snoring and daytime sleepiness are thought to stem from enhanced muscle responsiveness, increased upper airway muscle strength, and subsequent reductions in inspiratory flow limitations and arousals [12]. Guimaraes et al. observed a significant reduction in the AHI during rapid eye movement (REM) sleep stages, suggesting that orofacial myofunctional therapy may contribute to sustained increases in pharyngeal dilator tone, particularly the genioglossus muscle, across all sleep stages [13].

The difference in mechanism between myofascial exercises and regular breathing exercises, particularly in their application to OSA, is crucial in understanding their respective impacts. Myofascial exercises involve targeted movements aimed at strengthening facial muscles around the throat, jaw, and soft palate. By enhancing muscle tone and control in these specific areas, myofascial exercises aim to improve airway patency and reduce obstruction during sleep. In contrast, regular breathing exercises focus on enhancing overall respiratory function through techniques such as diaphragmatic breathing or paced breathing patterns. These exercises aim to optimize lung capacity, strengthen respiratory muscles, and promote relaxation, which can indirectly improve breathing patterns and reduce the effort required for breathing during sleep. The mechanisms differ fundamentally: myofascial exercises target anatomical factors directly related to airway collapse, while regular breathing exercises primarily enhance respiratory muscle function and coordination, potentially improving sleep quality in individuals with OSA. Understanding these distinct mechanisms is essential for tailoring effective therapeutic strategies for managing OSA and improving patient outcomes.

The study holds significant clinical relevance by highlighting the effectiveness of myofascial exercises and voluntary breathing techniques in managing sleep-disordered breathing issues in adolescents. By discerning which intervention might be more effective for certain individuals, it provides valuable guidance for tailoring treatments to meet individual needs. Moreover, the research findings can inform health policies, advocating for the integration of nonpharmacological interventions into the standard care for adolescent sleep disorders. Looking forward, this study's insights can direct future research endeavors toward investigating the long-term effects and wider applications of myofascial exercise and voluntary breathing techniques within the field of sleep medicine.

The study had limitations that could impact the generalizability and robustness of the findings. First, the relatively small sample size limits the statistical power to detect subtle differences between the interventions and may affect the representativeness of the results. Additionally, the short follow-up period may not adequately capture the long-term adherence and sustained impact of myofascial exercises and voluntary breathing techniques on the AHI and sleep quality. This underscores the need for longitudinal studies to better assess the durability of these interventions. Moreover, the study did not control for variables such as diet, physical activity, and preexisting conditions, which could influence sleep quality and AHI.

## Conclusions

In conclusion, our study demonstrates that both myofascial exercise and voluntary breathing techniques show promise in improving outcomes related to sleep-disordered breathing among adolescents. These findings suggest that incorporating myofascial exercise or voluntary breathing techniques into treatment regimens for adolescents with sleep-disordered breathing can lead to notable enhancements in AHI and sleep quality. Further research is warranted to elucidate the long-term effectiveness and broader applicability of these interventions.
